# Metal–Organic Frameworks (MOFs) for Cancer Therapy

**DOI:** 10.3390/ma14237277

**Published:** 2021-11-28

**Authors:** Mohammad Reza Saeb, Navid Rabiee, Masoud Mozafari, Francis Verpoort, Leonid G. Voskressensky, Rafael Luque

**Affiliations:** 1 Department of Polymer Technology, Faculty of Chemistry, Gdánsk University of Technology, G. Narutowicza 11/12, 80-233 Gdánsk, Poland; mrsaeb2008@gmail.com; 2 Department of Physics, Sharif University of Technology, Tehran P.O. Box 11155-9161, Iran; 3 School of Engineering, Macquarie University, Sydney, NSW 2109, Australia; 4 Lunenfeld-Tanenbaum Research Institute, Mount Sinai Hospital, University of Toronto, Toronto, ON M5G 1X5, Canada; m.mozafari@utoronto.ca; 5 Laboratory of Organometallics, Catalysis and Ordered Materials, State Key Laboratory of Advanced Technology for Materials Synthesis and Processing, Wuhan University of Technology, Wuhan 430070, China; francis.verpoort@ghent.ac.kr; 6 National Research Tomsk Polytechnic University, Lenin Avenue 30, 634050 Tomsk, Russia; 7 Global Campus Songdo, Ghent University, 119 Songdomunhwa-Ro, Ywonsu-Gu, Incheon 21985, Korea; 8 Department of Chemistry, Peoples Friendship University of Russia (RUDN University), 6 Miklukho-Maklaya Str., 117198 Moscow, Russia; lvoskressensky@yandex.ru; 9 Departamento de Química Orgánica, Universidad de Córdoba, Campus de Rabanales, Edificio Marie Curie (C-3), Ctra Nnal IV-A, Km 396, E14014 Cordoba, Spain

**Keywords:** metal-organic frameworks (MOFs), cancer therapy, biotechnology, nanomedicine

## Abstract

MOFs exhibit inherent extraordinary features for diverse applications ranging from catalysis, storage, and optics to chemosensory and biomedical science and technology. Several procedures including solvothermal, hydrothermal, mechanochemical, electrochemical, and ultrasound techniques have been used to synthesize MOFs with tailored features. A continued attempt has also been directed towards functionalizing MOFs via “post-synthetic modification” mainly by changing linkers (by altering the type, length, functionality, and charge of the linkers) or node components within the MOF framework. Additionally, efforts are aimed towards manipulating the size and morphology of crystallite domains in the MOFs, which are aimed at enlarging their applications window. Today’s knowledge of artificial intelligence and machine learning has opened new pathways to elaborate multiple nanoporous complex MOFs and nano-MOFs (NMOFs) for advanced theranostic, clinical, imaging, and diagnostic purposes. Successful accumulation of a photosensitizer in cancerous cells was a significant step in cancer therapy. The application of MOFs as advanced materials and systems for cancer therapy is the main scope beyond this perspective. Some challenging aspects and promising features in MOF-based cancer diagnosis and cancer therapy have also been discussed.

## 1. MOFs for Cancer Therapy: So Far, So Close!

**Figure 1:**
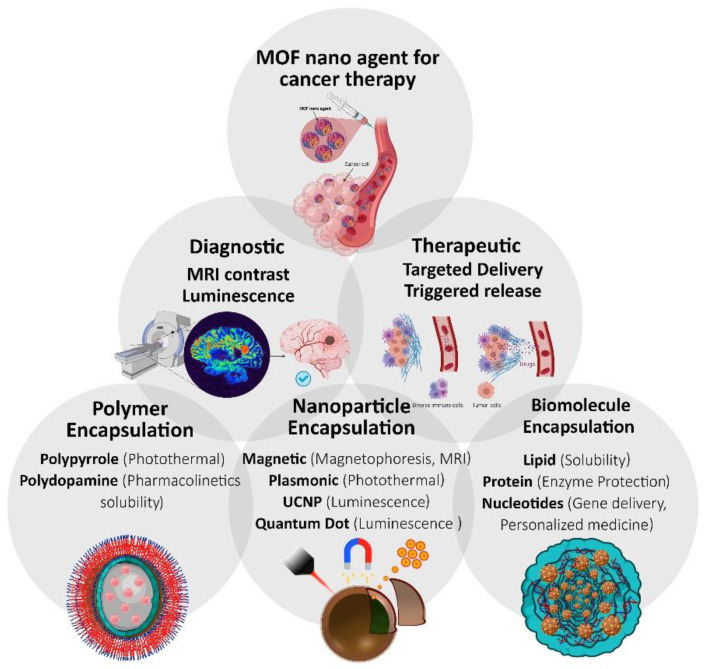
Main scope of this perspective regarding the use of MOFs-based nanomaterials for advanced cancer therapy.

Scientists are continuously seeking new types of treatments, early diagnosis, and early detection in order to combat diseases such as cancer [1,2,3]. The potential of MOFs as advanced materials and systems for cancer therapy is the main scope beyond this perspective [4,5,6]. Some challenging aspects and promising features in MOF-based cancer diagnosis and cancer therapy are also discussed (Figure 1). This is an interesting field of science with progressive advancements that need much focused attention in order to make a full transition from bench to bedside; however, till now, there are limited successful case studies able to provide a phase change to clinics. This is an important aspect to diagnose the limitations and inhibiting factors regarding the use of advanced materials including MOFs for therapy for cancer progressing to clinical stages.

## 2. MOFs in Detecting Cancer Biomarkers

Imaging and photofunctional technologies are rapidly developing to provide scientists with a visual tool for cancer diagnosis. Biological markers, or briefly biomarkers, function as an index to quantitatively express the state of the biological microenvironments for the sake of detection and diagnostic purposes [7,8]. Medical therapeutic approaches benefiting from biomarkers work on the basis of blood, urine, or soft tissues. The use of biomarkers makes possible the examination of biological processes working properly such as pharmacologic response of soft tissue to a therapeutic protocol [9,10]. In other words, biomarkers enable medical researchers and doctors to find and deepen the understanding of an interrelationship between the risk of human disease and a therapeutic protocol [11,12,13,14,15]. Indeed, high surface area and tunable micro- and nanoporous structure are key enabling features in the use of MOFs as biomarkers (Table 1; classification of MOF applications in bioimaging). In this regard, efficient recognition of elements is prioritized in MOFs optimization. Additional features including high thermal stability would be necessary, e.g., applying hyperthermia in cancer immunotherapy. MOFs are widely employed for luminescence sensing or photofunctionality as well as chemical detection of species in cancer therapy. MOFs’ inherently induced optical and photonic properties are fueled by both organic ligands and metal ions and dyes or markers encapsulated in MOFs. Correspondingly, mechanisms including metal-to-ligand or ligand-to-metal charge transfer as well as ligand-ligand and metal-metal charge transfer are liable for luminescence emission.

A variety of nanoparticles hybridized with MOFs have been developed for chemical sensing. For example, lanthanide-functionalized MOFs are exceptional structures, each representing a specific luminescence color, all detectable in the visible region. Each lanthanide has its distinctive signatures to be taken into account for a targeted detection mission. Manipulation of porosity gives rise to the development of complex biomarkers for early detection of the tumor as well as visual monitoring of anticancer drug loading and release. Detection of nucleic acids and proteins and small physiological molecules is known as a route for MOF-based cancer diagnosis. For example, ovarian and some gynecological cancers can be diagnosed at early stages by exploring a correlation between fluorescence intensity and dosage of lysophosphatidic acid as a biomarker [15].

## 3. MOFs for Enhanced Cancer Therapy

Cancer is a complex phenomenon arising from RNA damage. Effective and rational cancer therapy is pertinent to the degree of success in understanding the mechanisms controlling the regeneration and proliferation of cancerous cells [27,28,29]. Targeted cancer therapy seeks to address the causation and visualize the generation and distribution of the cancerous cells [30,31,32]. The focus in targeted therapeutics is placed on exploring highly efficient noninvasive pathways to make it possible to precisely attack the region from which cancer cells are generated and proliferated [33]. Nanomedicine, the use of nanoparticles in medicine, makes good use of nanochemotherapeutics for cancer treatment. This field has been experiencing a progressive growth period since the early 21st century. Attention has been paid to treating cancer by changing attitudes in a worldwide shift from disparate to clinical investigations. Because of the aforementioned beneficial features, MOFs are widely used as tailorable theranostic platforms for both cancer diagnosis and cancer treatment, including monomodal therapeutics such as photodynamic therapy (PDT), photothermal therapy (PTT), chemotherapy, radiography, and immunotherapy, as well as multimodal/combined imaging, thermal, and chemotropic treatments [34]. An overview of the literature on the use of MOF in individual cancer therapy is summarized in Table 2.

PDT-based treatment works on the bedrock of administration of a photosensitizer supported via irradiation of cancerous cells at a wavelength in the vicinity of the absorbance band of the sensitizing agent. It is a clinically approved therapeutic with a very low possibility of invasion. PTT in the oxygen atmosphere enables one to directly attack the tumor cells and induces a local inflammatory reaction, which appears promising at the early stages of cancer [61]. MOFs are successfully applied in PDT in vivo. The strategy is based on modification and/or functionalization of MOFs to make them photosensitizers working efficiently under a specified laser irradiation wavelength. For example, in situ polymerization of dopamine with Mn, Co, or UiO-66 frameworks resulted in hybrid photosensitizing agents inducing cancer cell apoptosis [62,63]. A wide variety of MOFs, including Pt-MOF, Co(Hmim)_2_ (ZIF-67), Coordination Polymer of Oslo (CPO-27)–M (M = Zn, Ni, and Mg), Hong Kong University of Science and Technology (HKUST)-1, Fe– Materials Institute Lavoisier (MIL)-101–NH_2_, Universitetet i Oslo (UiO)-66–NH_2_, and Isoreticular Metal-organic Framework (IRMOF)-3 possessing absorption bands in the range of light emission have been applied as photosensitizing agents in cancer therapy [64]. For instance, UiO-66@PAN, possessing uniform size and dispersibility in aqueous solution, is localized via endocytosis and revealed excellent PTT effect in vitro, significantly inhibiting colon cancers’ growth in vivo [48].

Chemotherapeutic techniques, with and without radiography as their complements, have been reported in literature. Nevertheless, clinical data seems necessary to assess their efficiency compared to PTT and PDT techniques. For instance, in bowel cancer, chemotherapy could very limitedly be supported by radiotherapy, to reduce locoregional relapse [65]. A core/shell, namely persistent luminescence-sensitive UiO-66 NMOFs hybridized with mesoporous carbon, is applied in imaging-guided chemotherapy. Particle size is as small as ca 70 nm with a tunable pore size (∼4.8–16.2 nm) in the shell and NIR luminescence from the core. Three model drugs used revealed enhanced delivery and tumor therapy [66]. The encapsulation of Adriamycin as a model anticancer drug in Zn-MOF hybridized with folic acid resulted in the development of a promising drug delivery system (DDS) as well as tumor-targeted chemotherapy of cervical cancer, as evidenced both in vitro and in vivo [67].

Application of MOFs for bone cancer therapy is based on radiotherapy-accelerated tumor ablation and prevention of lung metastasis, which is featured by the reduction in hypoxia-inducible factor [68]. A combined radiotherapy/radiodynamic therapy with the aid of NMOFs ended in a successful clinical treatment by eliminating lung metastases, which resulted from reactivating antitumor immunity and inhibiting myeloid-derived suppressor cells [69]. MOFs are also used in immunotherapy of cancer, individually or in combination with other therapeutics. Site-specific ZIF-8 modified with hyaluronic acid resulted in enhanced immunotherapy, particularly when aided by photothermal agents, but still, the activation of immune response was inadequate [70]. To overcome such a low efficiency in immunotherapy, lysosome-targeting Zn-NMOFs coupled with a lysosome-targeting aptamer (CD63-aptamer) with pH-sensitive character and high capacity for protein encapsulation are examined with improved antitumor effect of T cells [71]. Some RNA interference (RNAi) techniques benefit from sequence-specific and/or post-transcriptional gene silencing to regulate the expression of proteins.

When used individually, cancer therapeutics can kill cancer cells limitedly. The combination of two or more therapeutics reduces side effects and synergistically enhances anticancer efficacy. MOF-based combined therapeutic methods such as chemophototherapy or PTT/PDT have also been carried out in order to increase the effectiveness of treatments. For example, highly tailorable core/shell nanoplatforms based on porphyrinic MOF-coated gold nanorods used in PTT/PDT/chemotherapy enhanced drug-loading capacity as well as showed near-infrared (NIR) light sensitivity for imaging; moreover, they generated reactive oxygen and provided the tumor with photothermal activity in cancer treatment [72]. There have been some more complex cases such as dual-mode and photoacoustic imaging as well as gas therapy combined with PTT and chemotherapy based on Fe (Ⅲ)-based NMOF (MIL-100) nanocarriers used by the NIR-based drug/gene delivery to kill cancer cells [73,74]. Stimuli-responsive hybrid NMOFs with precisely tailored structure and modulated release are also used successfully in treating breast cancer cells by applying combined PDT/chemotherapy [75].

## 4. Multifunctional MOFs for Cancer Theranostics

Over the past decade, a great deal of attention has been directed to the modification and functionalization of NMOFs through different methods to enhance their therapeutic efficiency. In this regard, multifunctional NMOFs are developed which rely on targeted cancer therapy. Surface modification of MOFs occurring in the course of self-assembly and post-synthetically occurring modifications are two major classes that trigger the outside surface of MOFs. Polymer-coated or polymer-wrapped MOFs are a class of functionalized MOFs by which one can enlarge the biomedical application window of NMOFs. Attachment of polymers possessing reactive groups like carbohydrate polymers to MOFs leads to enhanced stability and dispersion. For instance, UiO-66 MOFs functionalized with biomacromolecules follow an enhanced cell endocytosed mechanism. NMOFs can also be polymer functionalized by GraftFast methodology, e.g., PEGylated MIL-100(Fe); besides, the radical polymerization mechanism is frequently applied in developing polymer-wrapper NMOFs [76]. Molecularly imprinted polymer (MIP)-coated MOFs with on/off luminescent behavior have been developed for the selective detection of species in biological environments. Several MIP-wrapped NMOFs have also been developed as DDS, representing controlled release behavior and merit for oral administration [77].

Magnetic bio-MOFs are a broad class of NMOFs employed for cancer therapy. This group of NMOFs can be distinguished based on its mechanism, which supports higher relaxivity and enhanced sensitivity in MRI with respect to unmodified magnetic (MOF-free) nanoparticles. There are several examples, including gold-incorporated MOFs for magnetic resonance imaging (MRI) and PTT for breast cancer treatment [78], target-specific anticancer ZIF-8/enzyme hybrid MOFs with minimal damage to the healthy cells [79], Fe_3_O_4_@bio-MOF-folic-acid-chitosan conjugate (FC) hybrid structures as theranostic in breast cancer [80], Zr-MOF@glucose-6-phosphate applied in kidney cancer treatment [81], highly selective and sensitive Cu-MOFs for liver cancer therapy [82], Fe_3_O_4_@nickel-cadmium quantum dots (QDs)/MOFs as a biosensor for prostate cancer diagnosis [83], and Fe_3_O_4_@5-aminolevulinic-acid-Zn MOF for MRI and brain tumor therapy [84]. There are also several disparate papers on the design, synthesis, and application of complex core-shell MOFs and flexible MOF-based theranostic nanoplatforms, mainly for multimodal imaging technologies.

## 5. Conclusions, Challenging Features, and Future Perspectives

MOFs are highly porous biocompatible tailorable hybrid structures with therapeutic effects on cancerous cells and tumors as a result of their ability to encapsulate cargos (drugs, proteins, genes, etc.). The use of MOFs in cancer therapy is experiencing an early stage of development because their immune response activation is still inadequate for efficient cancer treatment. Despite the potential of using a bewildering array of materials and elements (individually or in the form of core/shell, hybrid, and functionalized multiple structures) to be employed in MOF synthesis, optimizing such structures for targeted therapy requires the examination of a variety of scenarios. This challenge is, in a complex manner, compounded with the inadequate efficacy of drugs encapsulated in MOFs when it comes to being encapsulated in large amounts within the framework, and difficulties associated with targeting cargos under modulated release rate at the cancerous zone.

In other words, therapeutic drugs can partially attack tumor sites; moreover, distinguishing cancer cells from normal cells without damage to the healthy tissues and organs is not easily achievable. The use of NOMFs to an acceptable level compensates for such an inability. Traditional therapeutics such as radiotherapy and chemotherapy principally suffer from an uncontrolled attack on cancer cells. In contrast, phototherapeutics, to a large extent, are targeted at cancer sites. PTT and PDT techniques are widely used in cancer therapy, while chemotherapy, radiotherapy, and combined methodologies have also been implemented to treat cancer, mainly bone, breast, and colon cancers. Nevertheless, a long road must be traveled to overcome the limited translation of NMOFs to clinical therapies by designing target-specific tailored NMOFs for targeted actions, which we hope to witness in the near future.

## Figures and Tables

**Table 1:** MOF classification for bioimaging and related applications.

Type of MOF	Imaging Method and Biomedical Application	Ref.
UCNP@Fe-MIL-101-NH_2_	Optical Imaging (OI)/magnetic resonance imaging (MRI)- Cancer therapy- Tumor imaging	[16]
DOX@NPMOFs	OI- Tumor imaging- Cancer diagnosis- Cancer therapy	[17]
DOX@Gd-MOFs-Glu	Computed tomography (CT)/MRI- Cancer therapy- Tumor imaging- Targeted delivery of cancer drug	[18]
TPZ/Hf/TCPP/PEG	CT—Cancer therapy- Tumor imaging- Targeted delivery of cancer drug	[19]
Eu, Gd-NMOF@SiO_2_	MRI- Cancer therapy- Tumor imaging	[20]
Fe_3_O_4_@IFMOF-3/FA	MRI- Cancer therapy- Tumor imaging	[21]
UiO-66@DOPA-LB	OI- Tumor imaging- Cancer diagnosis- Cancer therapy	[22]
Fe_3_O_4_-ZIF-8	MRI- Cancer therapy- Tumor imaging- Early detection of tumor sites	[23]
MOF@HA@ICG NPs	MRI- Cancer therapy- Tumor imaging- Early detection of tumor sites	[24]
Au@MIL-88 (Fe)	CT/MRI- Cancer therapy- Tumor imaging- Targeted delivery of cancer drug	[25]
^89^Zr-UiO-66/Py-PGA-PEG-F3	Positron emission tomography (PET) imaging- Cancer therapy- Tumor imaging- Targeted delivery of cancer drug	[26]

**Table 2:** A literature survey on NMOFs in individual cancer therapy.

Method	NMOFs	In Vitro Cell Lines	In Vivo Models	Ref.
Chemotherapy	Cisplatin@NMOF-1/DOX@NMOF-1	HeLa	-	[35]
	DOX@NMOF-VEGF responsive	MDA-MB-231	-	[36]
	ZIF-8/FA@UCNP	HeLa	-	[37]
	UiO-67/UiO-66	U-87 MG/HSC-3	-	[38]
	Fe-MIL-53-NH_2_-FA-5-FAM@5-FU	MGC-803	-	[39]
	UiO-68-FA@DOX	HepG2	Mice with HepG2 tumors	[40]
	Gd-MOF-Glu@DOX	HeLa	Mice with HeLa tumors	[18]
	IRMOF-3@Fe_3_O_4_/FA	Hea	-	[21]
	ZIF-8@P	MDA-MB-231	-	[41]
	ZIF-8@Fe_3_O_4_	MCF-7	-	[42]
	^89^Zr-UiO-66@Py-PGA-PEG@F3	MDA-MB-231	Mice with MDA-MB-231 tumors	[26]
	Fe_3_O_4_@IFMOF-3@OCMP@FA	HeLa	-	[43]
	DPB-UiO-based NMOFs	HeLa, MCF-7 and etc.	Mice with HeLa and MCF-7 tumors	[44,45]
RT-RDT	W_18_@Hf_12_-DBB-Ir	MC38/CT26	Mice with MC38/CT26 tumors	[46]
	DBB-Ru-Hf	MC38/CT26	Mice with MC38/CT26 tumors	[47]
PTT	UiO-66@PAN	CT26/HCT116	Mice with CT26 tumors	[48]
	Mn-IR822@PEG-PDA	4T1	Mice with 4T1 tumors	[49]
	MOF@ICG@HA	MCF-7	Mice with MCF-7 tumors	[24]
PDT	Ti-TBP	CT26	Mice with CT26 tumors	[50]
	PCN-FA-224	A549/HeLa	-	[51]
	UiO-DBC	HT29/CT26	Mice with HT29/CT26 tumors	[52]
	MB@THA-MOF-76@cRGD	A549	-	[53]
	MOF-FA@PS	HeLa	-	[54]
	UiO-DBP	SQ20B	Mice with SQ20B tumors	[55]
	PCN-224 (Pt)	4T1/HeLa	Mice with H22 tumors	[56]
	NP-1	HCT116/HepG2	Mice with HCT116 tumors	[57]
	ZnDTPP-I_2_@UiO-66	HepG2	-	[58]
	TPP-SH@UiO-66	HeLa	-	[59]
	Ru(bpy)_3_^2+^@(UiO-67)	A549	-	[60]

## Data Availability

Not applicable.

## Abbreviations

Metal-organic frameworks: MOFs; nano-MOFs: NMOFs; magnetic resonance imaging: MRI; optical imaging: OI; computed tomography: CT; positron emission tomography: PET; photodynamic therapy: PDT; photothermal therapy: PTT.

## References

[1] Hajebi S., Rabiee N., Bagherzadeh M., Ahmadi S., Rabiee M., Roghani-Mamaqani H., Tahriri M., Tayebi L., Hamblin M.R. (2019). Stimulus-responsive polymeric nanogels as smart drug delivery systems. Acta Biomater..

[2] Rabiee N., Ahmadi S., Afshari R., Khalaji S., Rabiee M., Bagherzadeh M., Fatahi Y., Dinarvand R., Tahriri M., Tayebi L. (2021). Polymeric Nanoparticles for Nasal Drug Delivery to the Brain: Relevance to Alzheimer's Disease. Adv. Ther..

[3] Rabiee N., Ahmadvand S., Ahmadi S., Fatahi Y., Dinarvand R., Bagherzadeh M., Rabiee M., Tahriri M., Tayebi L., Hamblin M.R. (2020). Carbosilane dendrimers: Drug and gene delivery applications. J. Drug Deliv. Sci. Technol..

[4] Rabiee N., Yaraki M.T., Garakani S.M., Garakani S.M., Ahmadi S., Lajevardi A., Bagherzadeh M., Rabiee M., Tayebi L., Tahriri M. (2020). Recent advances in porphyrin-based nanocomposites for effective targeted imaging and therapy. Biomaterials.

[5] Rabiee N., Bagherzadeh M., Heidarian Haris M., Ghadiri A.M., Matloubi Moghaddam F., Fatahi Y., Dinarvand R., Jarahiyan A., Ahmadi S., Shokouhimehr M. (2021). Polymer-Coated NH2-UiO-66 for the Codelivery of DOX/pCRISPR. ACS Appl. Mater. Interfaces.

[6] Rabiee N., Bagherzadeh M., Jouyandeh M., Zarrintaj P., Saeb M.R., Mozafari M., Shokouhimehr M., Varma R.S. (2021). Natural Polymers Decorated MOF-MXene Nanocarriers for Co-Delivery of Doxorubicin/pCRISPR. ACS Appl. Bio Mater..

[7] Banerjee S., Lollar C.T., Xiao Z., Fang Y., Zhou H.-C. (2020). Biomedical integration of metal–organic frameworks. Trends Chem..

[8] Saeb M.R., Rabiee N., Mozafari M., Mostafavi E. (2021). Metal-organic frameworks-based nanomaterials for drug delivery. Materials.

[9] Rabiee N., Bagherzadeh M., Ghadiri A.M., Salehi G., Fatahi Y., Dinarvand R. (2020). ZnAl nano layered double hydroxides for dual functional CRISPR/Cas9 delivery and enhanced green fluorescence protein biosensor. Sci. Rep..

[10] Rabiee N., Bagherzadeh M., Ghadiri A.M., Fatahi Y., Baheiraei N., Safarkhani M., Aldhaher A., Dinarvand R. (2021). Bio-multifunctional noncovalent porphyrin functionalized carbon-based nanocomposite. Sci. Rep..

[11] Rabiee N., Fatahi Y., Asadnia M., Daneshgar H., Kiani M., Ghadiri A.M., Atarod M., Mashhadzadeh A.H., Akhavan O., Bagherzadeh M. (2022). Green porous benzamide-like nanomembranes for hazardous cations detection, separation, and concentration adjustment. J. Hazard. Mater..

[12] Zare H., Ahmadi S., Ghasemi A., Ghanbari M., Rabiee N., Bagherzadeh M., Karimi M., Webster T.J., Hamblin M.R., Mostafavi E. (2021). Carbon Nanotubes: Smart Drug/Gene Delivery Carriers. Int. J. Nanomed..

[13] Nikfarjam N., Ghomi M., Agarwal T., Hassanpour M., Sharifi E., Khorsandi D., Ali Khan M., Rossi F., Rossetti A., Nazarzadeh Zare E. (2021). Antimicrobial ionic liquid-based materials for biomedical applications. Adv. Funct. Mater..

[14] Bagherzadeh M., Rabiee N., Fatahi Y., Dinarvand R. (2021). Zn-rich (GaN)1−x (ZnO)x: A biomedical friend?. New J. Chem..

[15] Yan B. (2019). Photofunctional MOF-based hybrid materials for the chemical sensing of biomarkers. J. Mater. Chem. C.

[16] Li Y., Tang J., He L., Liu Y., Liu Y., Chen C., Tang Z. (2015). Core–Shell Upconversion Nanoparticle@ Metal–Organic Framework Nanoprobes for Luminescent/Magnetic Dual-Mode Targeted Imaging. Adv. Mater..

[17] Liu W., Wang Y.M., Li Y.H., Cai S.J., Yin X.B., He X.W., Zhang Y.K. (2017). Fluorescent imaging-guided chemotherapy-and-photodynamic dual therapy with nanoscale porphyrin metal–organic framework. Small.

[18] Zhang H., Shang Y., Li Y.-H., Sun S.-K., Yin X.-B. (2018). Smart metal–organic framework-based nanoplatforms for imaging-guided precise chemotherapy. ACS Appl. Mater. Interfaces.

[19] Liu M., Wang L., Zheng X., Liu S., Xie Z. (2018). Hypoxia-triggered nanoscale metal–organic frameworks for enhanced anticancer activity. ACS Appl. Mater. Interfaces.

[20] Wang G.D., Chen H., Tang W., Lee D., Xie J. (2016). Gd and Eu co-doped nanoscale metal–organic framework as a T1–T2 dual-modal contrast agent for magnetic resonance imaging. Tomography.

[21] Chowdhuri A.R., Bhattacharya D., Sahu S.K. (2016). Magnetic nanoscale metal organic frameworks for potential targeted anticancer drug delivery, imaging and as an MRI contrast agent. Dalton Trans..

[22] Zhang R., Qiao C., Jia Q., Wang Y., Huang H., Chang W., Wang H., Zhang H., Wang Z. (2019). Highly Stable and Long-Circulating Metal-Organic Frameworks Nanoprobes for Sensitive Tumor Detection In Vivo. Adv. Healthc. Mater..

[23] Lin J., Xin P., An L., Xu Y., Tao C., Tian Q., Zhou Z., Hu B., Yang S. (2019). Fe3O4–ZIF-8 assemblies as pH and glutathione responsive T2 –T1 switching magnetic resonance imaging contrast agent for sensitive tumor imaging in vivo. Chem. Commun..

[24] Cai W., Gao H., Chu C., Wang X., Wang J., Zhang P., Lin G., Li W., Liu G., Chen X. (2017). Engineering phototheranostic nanoscale metal–organic frameworks for multimodal imaging-guided cancer therapy. ACS Appl. Mater. Interfaces.

[25] Shang W., Zeng C., Du Y., Hui H., Liang X., Chi C., Wang K., Wang Z., Tian J. (2017). Core–shell gold Nanorod@ metal–organic framework nanoprobes for multimodality diagnosis of glioma. Adv. Mater..

[26] Chen D., Yang D., Dougherty C.A., Lu W., Wu H., He X., Cai T., Van Dort M.E., Ross B.D., Hong H. (2017). In vivo targeting and positron emission tomography imaging of tumor with intrinsically radioactive metal–organic frameworks nanomaterials. ACS Nano.

[27] Rabiee N., Khatami M., Jamalipour Soufi G., Fatahi Y., Iravani S., Varma R.S. (2021). Diatoms with Invaluable Applications in Nanotechnology, Biotechnology, and Biomedicine: Recent Advances. ACS Biomater. Sci. Eng..

[28] Rabiee N., Bagherzadeh M., Ghadiri A.M., Fatahi Y., Aldhaher A., Makvandi P., Dinarvand R., Jouyandeh M., Saeb M.R., Mozafari M. (2021). Turning Toxic Nanomaterials into a Safe and Bioactive Nanocarrier for Co-Delivery of DOX/pCRISPR. ACS Appl. Bio Mater..

[29] Rabiee N., Bagherzadeh M., Ghadiri A.M., Kiani M., Fatahi Y., Tavakolizadeh M., Pourjavadi A., Jouyandeh M., Saeb M.R., Mozafari M. (2021). Multifunctional 3D hierarchical bioactive green carbon-based nanocomposites. ACS Sustain. Chem. Eng..

[30] Saeb M.R., Rabiee N., Seidi F., Far B.F., Bagherzadeh M., Lima E.C., Rabiee M. (2021). Green CoNi2S4/Porphyrin Decorated Carbon-based Nanocomposites for Genetic Materials Detection. J. Bioresour. Bioprod..

[31] Rabiee N., Bagherzadeh M., Ghadiri A.M., Kiani M., Ahmadi S., Jajarmi V., Fatahi Y., Aldhaher A., Tahriri M., Webster T.J. (2021). Calcium-based nanomaterials and their interrelation with chitosan: Optimization for pCRISPR delivery. J. Nanostruct. Chem..

[32] Zarghami Dehaghani M., Yousefi F., Sajadi S.M., Tajammal Munir M., Abida O., Habibzadeh S., Mashhadzadeh A.H., Rabiee N., Mostafavi E., Saeb M.R. (2021). Theoretical Encapsulation of Fluorouracil 5-FU. Anti-Cancer Chemotherapy Drug into Carbon Nanotubes CNT. and Boron Nitride Nanotubes BNNT. Molecules.

[33] Sawyers C. (2004). Targeted cancer therapy. Nature.

[34] Yang J., Yang Y.W. (2020). Metal-organic framework-based cancer theranostic nanoplatforms. View.

[35] Samanta D., Roy S., Sasmal R., Saha N.D., Viswanatha R., Agasti S.S., Maji T.K. (2019). Solvent Adaptive Dynamic Metal-Organic Soft Hybrid for Imaging and Biological Delivery. Angew. Chem..

[36] Chen W.-H., Sung S.Y., Fadeev M., Cecconello A., Nechushtai R., Willner I. (2018). Targeted VEGF-triggered release of an anti-cancer drug from aptamer-functionalized metal–organic framework nanoparticles. Nanoscale.

[37] Chowdhuri A.R., Laha D., Pal S., Karmakar P., Sahu S.K. (2016). One-pot synthesis of folic acid encapsulated upconversion nanoscale metal organic frameworks for targeting, imaging and pH responsive drug release. Dalton Trans..

[38] Filippousi M., Turner S., Leus K., Siafaka P.I., Tseligka E.D., Vandichel M., Nanaki S.G., Vizirianakis I.S., Bikiaris D.N., Van Der Voort P. (2016). Biocompatible Zr-based nanoscale MOFs coated with modified poly (ε-caprolactone) as anticancer drug carriers. Int. J. Pharm..

[39] Gao X., Zhai M., Guan W., Liu J., Liu Z., Damirin A. (2017). Controllable synthesis of a smart multifunctional nanoscale metal–organic framework for magnetic resonance/optical imaging and targeted drug delivery. ACS Appl. Mater. Interfaces.

[40] Li Y.-A., Zhao X.-D., Yin H.-P., Chen G.-J., Yang S., Dong Y.-B. (2016). A drug-loaded nanoscale metal–organic framework with a tumor targeting agent for highly effective hepatoma therapy. Chem. Commun..

[41] Zhou W., Wang L., Li F., Zhang W., Huang W., Huo F., Xu H. (2017). Selenium-containing polymer@ metal-organic frameworks nanocomposites as an efficient multiresponsive drug delivery system. Adv. Funct. Mater..

[42] Zhuang J., Kuo C.-H., Chou L.-Y., Liu D.-Y., Weerapana E., Tsung C.-K. (2014). Optimized metal–organic-framework nanospheres for drug delivery: Evaluation of small-molecule encapsulation. ACS Nano.

[43] Chenot C.C., Robiette R.L., Collin S. (2019). First evidence of the cysteine and glutathione conjugates of 3-sulfanylpentan-1-ol in hop *Humulus lupulus* L.. J. Agric. Food Chem..

[44] Arun Kumar S., Balasubramaniam B., Bhunia S., Jaiswal M.K., Verma K., Khademhosseini A., Gupta R.K., Gaharwar A.K. (2021). Two-dimensional metal organic frameworks for biomedical applications. Wiley Interdiscip. Rev. Nanomed. Nanobiotechnol..

[45] Yang J., Yang Y.W. (2020). Metal–organic frameworks for biomedical applications. Small.

[46] Lan G., Ni K., Veroneau S.S., Luo T., You E., Lin W. (2019). Nanoscale metal–organic framework hierarchically combines high-Z components for multifarious radio-enhancement. J. Am. Chem. Soc..

[47] Ni K., Lan G., Veroneau S.S., Duan X., Song Y., Lin W. (2018). Nanoscale metal-organic frameworks for mitochondria-targeted radiotherapy-radiodynamic therapy. Nat. Commun..

[48] Wang W., Wang L., Li Y., Liu S., Xie Z., Jing X. (2016). Nanoscale polymer metal–organic framework hybrids for effective photothermal therapy of colon cancers. Adv. Mater..

[49] Yang Y., Liu J., Liang C., Feng L., Fu T., Dong Z., Chao Y., Li Y., Lu G., Chen M. (2016). Nanoscale metal–organic particles with rapid clearance for magnetic resonance imaging-guided photothermal therapy. ACS Nano.

[50] Lan G., Ni K., Veroneau S.S., Feng X., Nash G.T., Luo T., Xu Z., Lin W. (2019). Titanium-based nanoscale metal–organic framework for type I photodynamic therapy. J. Am. Chem. Soc..

[51] Park J., Jiang Q., Feng D., Mao L., Zhou H.-C. (2016). Size-controlled synthesis of porphyrinic metal–organic framework and functionalization for targeted photodynamic therapy. J. Am. Chem. Soc..

[52] Lu K., He C., Lin W. (2015). A chlorin-based nanoscale metal–organic framework for photodynamic therapy of colon cancers. J. Am. Chem. Soc..

[53] Jia J., Zhang Y., Zheng M., Shan C., Yan H., Wu W., Gao X., Cheng B., Liu W., Tang Y. (2018). Functionalized Eu (III)-based nanoscale metal–organic framework to achieve near-IR-triggered and-targeted two-photon absorption photodynamic therapy. Inorg. Chem..

[54] Zhang L., Lei J., Ma F., Ling P., Liu J., Ju H. (2015). A porphyrin photosensitized metal–organic framework for cancer cell apoptosis and caspase responsive theranostics. Chem. Commun..

[55] Lu K., He C., Lin W. (2014). Nanoscale metal–organic framework for highly effective photodynamic therapy of resistant head and neck cancer. J. Am. Chem. Soc..

[56] Zhang Y., Wang F., Liu C., Wang Z., Kang L., Huang Y., Dong K., Ren J., Qu X. (2018). Nanozyme decorated metal–organic frameworks for enhanced photodynamic therapy. ACS Nano.

[57] Ma Y., Li X., Li A., Yang P., Zhang C., Tang B. (2017). H2S-activable MOF nanoparticle photosensitizer for effective photodynamic therapy against cancer with controllable singlet-oxygen release. Angew. Chem..

[58] Zhou L.-L., Guan Q., Li Y.-A., Zhou Y., Xin Y.-B., Dong Y.-B. (2018). One-pot synthetic approach toward porphyrinatozinc and heavy-atom involved Zr-NMOF and its application in photodynamic therapy. Inorg. Chem..

[59] Kan J.-L., Jiang Y., Xue A., Yu Y.-H., Wang Q., Zhou Y., Dong Y.-B. (2018). Surface decorated porphyrinic nanoscale metal–organic framework for photodynamic therapy. Inorg. Chem..

[60] Chen R., Zhang J., Chelora J., Xiong Y., Kershaw S.V., Li K.F., Lo P.-K., Cheah K.W., Rogach A.L., Zapien J.A. (2017). Ruthenium (II) Complex Incorporated UiO-67 Metal–Organic Framework Nanoparticles for Enhanced Two-Photon Fluorescence Imaging and Photodynamic Cancer Therapy. ACS Appl. Mater. Interfaces.

[61] Agostinis P., Berg K., Cengel K.A., Foster T.H., Girotti A.W., Gollnick S.O., Hahn S.M., Hamblin M.R., Juzeniene A., Kessel D. (2011). Photodynamic therapy of cancer: An update. CA Cancer J. Clin..

[62] Wang D., Wu H., Zhou J., Xu P., Wang C., Shi R., Wang H., Wang H., Guo Z., Chen Q. (2018). In Situ One-Pot Synthesis of MOF–Polydopamine Hybrid Nanogels with Enhanced Photothermal Effect for Targeted Cancer Therapy. Adv. Sci..

[63] Zhang Y., Fu H., Chen S., Liu B., Sun W., Gao H. (2020). Construction of an iridium (III)-complex-loaded MOF nanoplatform mediated with a dual-responsive polycationic polymer for photodynamic therapy and cell imaging. Chem. Commun..

[64] Espín J., Garzón-Tovar L., Carné-Sánchez A., Imaz I., Maspoch D. (2018). Photothermal activation of metal–organic frameworks using a UV–vis light source. ACS Appl. Mater. Interfaces.

[65] Wolmark N., Wieand H.S., Hyams D.M., Colangelo L., Dimitrov N.V., Romond E.H., Wexler M., Prager D., Cruz A.B., Gordon P.H. (2000). Randomized trial of postoperative adjuvant chemotherapy with or without radiotherapy for carcinoma of the rectum: National Surgical Adjuvant Breast and Bowel Project Protocol R-02. J. Natl. Cancer Inst..

[66] Chen L.-J., Zhao X., Liu Y.-Y., Yan X.-P. (2020). Macrophage membrane coated persistent luminescence nanoparticle@ MOF-derived mesoporous carbon core–shell nanocomposites for autofluorescence-free imaging-guided chemotherapy. J. Mater. Chem. B.

[67] Sun J., Long X.-E., Li R., Hu C.-F., Ge X.-H. (2019). Adriamycin Loaded and Folic Acid Coated Zn-MOF for Tumor-Targeted Chemotherapy of Cervical Cancer. J. Biomater. Tissue Eng..

[68] Du C., Zhou M., Jia F., Ruan L., Lu H., Zhang J., Zhu B., Liu X., Chen J., Chai Z. (2021). D-arginine-loaded metal-organic frameworks nanoparticles sensitize osteosarcoma to radiotherapy. Biomaterials.

[69] Ni K., Lan G., Chan C., Duan X., Guo N., Veroneau S.S., Weichselbaum R.R., Lin W. (2019). Ultrathin metal-organic-layer mediated radiotherapy-radiodynamic therapy. Matter.

[70] Zhang H., Zhang J., Li Q., Song A., Tian H., Wang J., Li Z., Luan Y. (2020). Site-specific MOF-based immunotherapeutic nanoplatforms via synergistic tumor cells-targeted treatment and dendritic cells-targeted immunomodulation. Biomaterials.

[71] Zhao Q., Gong Z., Li Z., Wang J., Zhang J., Zhao Z., Zhang P., Zheng S., Miron R.J., Yuan Q. (2021). Target Reprogramming Lysosomes of CD8+ T Cells by a Mineralized Metal–Organic Framework for Cancer Immunotherapy. Adv. Mater..

[72] Zeng J.Y., Zhang M.K., Peng M.Y., Gong D., Zhang X.Z. (2018). Porphyrinic metal–organic frameworks coated gold nanorods as a versatile nanoplatform for combined photodynamic/photothermal/chemotherapy of tumor. Adv. Funct. Mater..

[73] Yao J., Liu Y., Wang J., Jiang Q., She D., Guo H., Sun N., Pang Z., Deng C., Yang W. (2019). On-demand CO release for amplification of chemotherapy by MOF functionalized magnetic carbon nanoparticles with NIR irradiation. Biomaterials.

[74] Wang Z., Sun Q., Liu B., Kuang Y., Gulzar A., He F., Gai S., Yang P., Lin J. (2021). Recent advances in porphyrin-based MOFs for cancer therapy and diagnosis therapy. Coord. Chem. Rev..

[75] Zhang L., Gao Y., Sun S., Li Z., Wu A., Zeng L. (2020). pH-Responsive metal–organic framework encapsulated gold nanoclusters with modulated release to enhance photodynamic therapy/chemotherapy in breast cancer. J. Mater. Chem. B.

[76] Forgan R.S. (2019). The surface chemistry of metal–organic frameworks and their applications. Dalton Trans..

[77] Zhang L.P., Mo C.E., Huang Y.P., Liu Z.S. (2019). Preparation of liquid crystalline molecularly imprinted polymer coated metal organic framework for capecitabine delivery. Part. Part. Syst. Charact..

[78] Zhang L., Liu C., Gao Y., Li Z., Xing J., Ren W., Zhang L., Li A., Lu G., Wu A. (2018). ZD2-Engineered Gold Nanostar@ Metal-Organic Framework Nanoprobes for T1-Weighted Magnetic Resonance Imaging and Photothermal Therapy Specifically Toward Triple-Negative Breast Cancer. Adv. Healthc. Mater..

[79] Li J., Li T., Gorin D., Kotelevtsev Y., Mao Z., Tong W. (2020). Construction and characterization of magnetic cascade metal-organic framework/enzyme hybrid nanoreactors with enhanced effect on killing cancer cells. Colloids Surf. A Physicochem. Eng. Asp..

[80] Nejadshafiee V., Naeimi H., Goliaei B., Bigdeli B., Sadighi A., Dehghani S., Lotfabadi A., Hosseini M., Nezamtaheri M.S., Amanlou M. (2019). Magnetic bio-metal–organic framework nanocomposites decorated with folic acid conjugated chitosan as a promising biocompatible targeted theranostic system for cancer treatment. Mater. Sci. Eng. C.

[81] Hu X., Wu Y., Deng C. (2020). Recognition of urinary N-linked glycopeptides in kidney cancer patients by hydrophilic carbohydrate functionalized magnetic metal organic framework combined with LC-MS/MS. Microchim. Acta.

[82] Sheta S.M., El-Sheikh S.M., Abd-Elzaher M.M., Salem S.R., Moussa H.A., Mohamed R.M., Mkhalid I.A. (2019). A novel biosensor for early diagnosis of liver cancer cases using smart nano-magnetic metal–organic framework. Appl. Organomet. Chem..

[83] Ehzari H., Amiri M., Safari M. (2020). Enzyme-free sandwich-type electrochemical immunosensor for highly sensitive prostate specific antigen based on conjugation of quantum dots and antibody on surface of modified glassy carbon electrode with core–shell magnetic metal-organic frameworks. Talanta.

[84] Ebrahimpour A., Alam N.R., Abdolmaleki P., Hajipour-Verdom B., Tirgar F., Ebrahimi T., Khoobi M. (2021). Magnetic Metal–Organic Framework Based on Zinc and 5-Aminolevulinic Acid: MR Imaging and Brain Tumor Therapy. J. Inorg. Organomet. Polym. Mater..

